# Patients with non-colorectal cancers may be at elevated risk of colorectal neoplasia

**DOI:** 10.7150/jca.40724

**Published:** 2020-03-04

**Authors:** Hamzah Abu-Sbeih, Faisal S. Ali, Wei Qiao, Phillip Lum, Mehnaz A. Shafi, Robert S. Bresalier, Ernest Hawk, Gottumukkala S. Raju, Yinghong Wang

**Affiliations:** 1 Department of Gastroenterology, Hepatology, and Nutrition, The University of Texas MD Anderson Cancer Center, Houston, Texas, USA.; 2 Department of Internal Medicine, Presence Saint Joseph Hospital, Chicago, IL, USA.; 3 Department of Biostatistics, The University of Texas MD Anderson Cancer Center, Houston, Texas, USA.; 4 Department of Clinical Cancer Prevention, The University of Texas MD Anderson Cancer Center, Houston, Texas, USA.

**Keywords:** colorectal neoplasia, colon adenoma, adenoma detection rate, colorectal cancer, screening, surveillance

## Abstract

**Background**: Screening for colonic neoplasia has decreased the incidence of colorectal cancer in the United States in the past two decades. Whether personal history of noncolorectal cancer is a risk factor for colonic neoplasia has not been well studied. We assessed the risk of colorectal neoplasia in noncolorectal cancer survivors.

**Methods**: We conducted a retrospective study of patients who had undergone colonoscopy for any indication between 2009 and 2018. Colonic adenoma detection rate and multivariate logistic regression were conducted to assess for the primary outcomes of the study.

**Results**: The study included 9408 cancer patients and 3295 control patients. Colonic adenomas were detected in 4503 cancer patients (48%) and 950 cancer-free patients (29%). Histologic examination of these adenomas revealed tubulovillous features in 620 patients (5%) and villous in 153 (1%). High-grade dysplasia was detected in 1611 patients (13%). Invasive colorectal adenocarcinoma was detected in 455 patients (12%); this rate was highest in patients with multiple myeloma (14%). Multivariate analysis revealed that a personal history of noncolorectal cancer was associated with increased risk of adenoma (Odd ratio, 2.04; 95% CI, 1.84-2.26; *P*<0.001). The adenoma detection rate was 30% in patients younger than 40 years (n=1211), 32% in patients between 41 and 50 years (n=812), 47% in patients between 51 and 60 years (n=2892), and 55% in patients older than 60 years (n=4493).

**Conclusions**: The adenoma detection rate in patients with a personal history of noncolorectal cancer is higher than the reported rate of the general population and our control group.

## Introduction

Colorectal cancer (CRC) is the second leading cause of cancer-related death among both men and women, with 14.5 deaths per 100,000 adults and a 5-year survival rate of 65.5% in the United States 1. The incidence of CRC in the United States has continued to decrease over the past 2 decades following the implementation of screening colonoscopies for colonic adenomatous polyps (CAP), which can be premalignant lesions, along with individual risk stratification for CRC 2,3. Colonoscopy allows for early detection and removal of CAP or early-stage CRC. Additionally, the advent of advanced endoscopic treatment for CAP, even large ones (>10 mm), has decreased the need for surgical intervention and lowered the morbidity and mortality associated with open intra-abdominal interventions 4. The implementation of CRC screening efforts, including colonoscopy, has recently been shown to be highly cost-effective over a patient's lifetime 5.

The current recommendations favor screening patients at average risk for CRC when they are 50 years old 6-8. When risk factors associated with early CRC are identified, such as a personal or family history of CRC, CAP, hereditary colonic polyposis syndromes, or inflammatory bowel disease, screening colonoscopies are initiated prior to 50 years of age 6,9. Several other risk factors for CRC have been proposed including obesity, smoking, male sex, heavy alcohol consumption, lack of physical activity, and lack of nutrients and vitamins 10,11. However, none of these proposed risk factors currently warrant early screening colonoscopies.

There has been concern regarding the possible association between noncolorectal cancers (NCRC) and CAP and CRC 50-52. The current body of evidence on this topic reveals conflicting results 12,13. The potential link between NCRC and CRC may be explained by a commonality in the genetic and molecular predispositions to cancer, particularly since a variety of cancers share the same cascade of events that lead to their progression from non-neoplastic tissue to a neoplastic process. CAP can progress to CRC by 2 different pathways involving inherited and acquired genetic mutations of the adenomatous polyposis coli (*APC*), *KRAS*, *TP53*, and *BRAF* genes 14-18. Additionally, microsatellite instability caused by alterations in DNA mismatch repair genes plays a pivotal role in the development of CRC 18 and has been identified in 27 other tumor types 19. Thus, associations between NCRC and CRC or its precursor, CAP, are possible and warrant further investigation. In our clinical practice at a tertiary cancer center, we noticed a high adenoma detection rate (ADR) among patients with personal history of NCRC. However, the current body of evidence addressing the risk of CRC or CAP after NCRC diagnosis is scarce.

The purpose of this study was to determine whether an association exists between NCRC and CRC or CAP. We also aimed to characterize the histologic features of CAP among patients at a tertiary cancer center with a history of NCRC.

## Methods and Materials

### Patient population

This retrospective study was conducted after obtaining approval from the institutional review board at The University of Texas MD Anderson Cancer Center. We included patients who had undergone their first colonoscopic evaluation for any indication between 2009 and 2018. Patients with a personal history of CRC, CAP, inflammatory bowel disease, or hereditary polyposis syndromes were excluded. Also, patients who underwent their first colonoscopy before the diagnosis of cancer were excluded. Patients were identified from endoscopy databases (EndoWorks, Center Valley, PA and later, Provation, Minneapolis, MN) at our institution using natural language processing 20. Then, data pertaining to this study were extracted from institutional databases.

### Clinical data

We obtained information regarding demographics and the presence, type, and treatment of cancer from the institutional tumor registry. Variables relating to medical history were collected from electronic medical charts. Demographics consisted of age, sex, race or ethnicity, and body mass index (BMI) at the time of the initial colonoscopy after cancer diagnosis. Family history of CRC in a first-degree relative and active or previous tobacco use were recorded. Patients were categorized into 2 groups, cancer or no-cancer, based on the presence of confirmed malignancy at the time of initial endoscopic screening. Cancer types were classified according to site, system, or cell lineage owing to the large number of cancer types, numerous classifications, and limited space to divide them further.

### Endoscopy and histology

In patients with polyps evident on colonoscopy, biopsies were taken to prove the presence of adenoma histologically. Endoscopic procedures with poor bowel preparation (Boston Bowel Preparation score lower than 2 for the examined regions of the colon) 21 or with severe colonic inflammation obscuring full examination of the colon were excluded. Histopathology reports were studied to document the presence of advanced adenoma features, such as villous or tubulovillous features or high-grade dysplasia, or advanced adenocarcinoma.

### Statistical analyses

Continuous variables were described using mean and standard deviation (SD) or median and interquartile range (IQR). Categorical variables were summarized using frequencies and percentages. Continuous variables were compared between 2 groups using the Wilcoxon rank sum test. Categorical variables were compared between groups using the Fisher exact or χ^2^ test. We performed multivariate logistic regression analysis to assess for risk factors associated with detection of CAP. Statistical tests were 2-sided. *P* values lower than 0.05 were considered significant. Statistical analyses were carried out using SAS version 9.4 (SAS Institute, Cary, NC) and SPSS version 24.0 (SPSS, Inc., Chicago, IL).

## Results

### Included patients

A total of 12703 patients had their first colonoscopy between 2009 and 2018. Colonoscopy was performed for CRC screening in 8252 patients, for GI symptoms in 3634, and for primary cancer assessment in 817. Most patients were female (55.1%). The mean age was 57 years (SD, 13 years). Most patients were white (68.2%), followed by black (10.8%) and Hispanic (9.0%). A history of smoking was reported in 37.4% of patients, and a family history of CRC was reported in 19.7%. The mean BMI at the time of first colonoscopy was 29.4 kg/m^2^ (SD, 6.6 kg/m^2^). A diagnosis of NCRC was present in 9408 patients, whereas 3295 had no cancer diagnosis. Clinical features of both groups are listed in **Table 1**.

### Patients with CAP

CAP was detected in 5453 (42.9%) patients: 4503 NCRC patients (48%) and 950 cancer-free patients (29%). Characteristics of patients with and without CAP are compared in **Table 2**. The median time from cancer diagnosis to CAP detection was 3 years (IQR, 1-8). On multivariate logistic regression analysis, the following factors were associated with increased risk of CAP: older age (odd ratio [OR], 1.03; 95% confidence interval [CI], 1.03-1.04; *P* < 0.001), male sex (OR, 1.49; 95% CI, 1.37-1.62; *P* < 0.001), family history of CRC (OR, 1.38; 95% CI, 1.25-1.54; *P* < 0.001), high BMI (OR, 1.03; 95% CI, 1.02-1.03; *P* < 0.001), and personal history of NCRC (2.04; 95% CI, 1.84-2.26; *P* < 0.001; **Table 3**). The ADR was 49.8% for patients who underwent colonoscopy for CRC screening and 30.2% for patients who underwent colonoscopy for other indications (*P* < 0.001).

### ADR by cancer type and age

**Figure 1:**
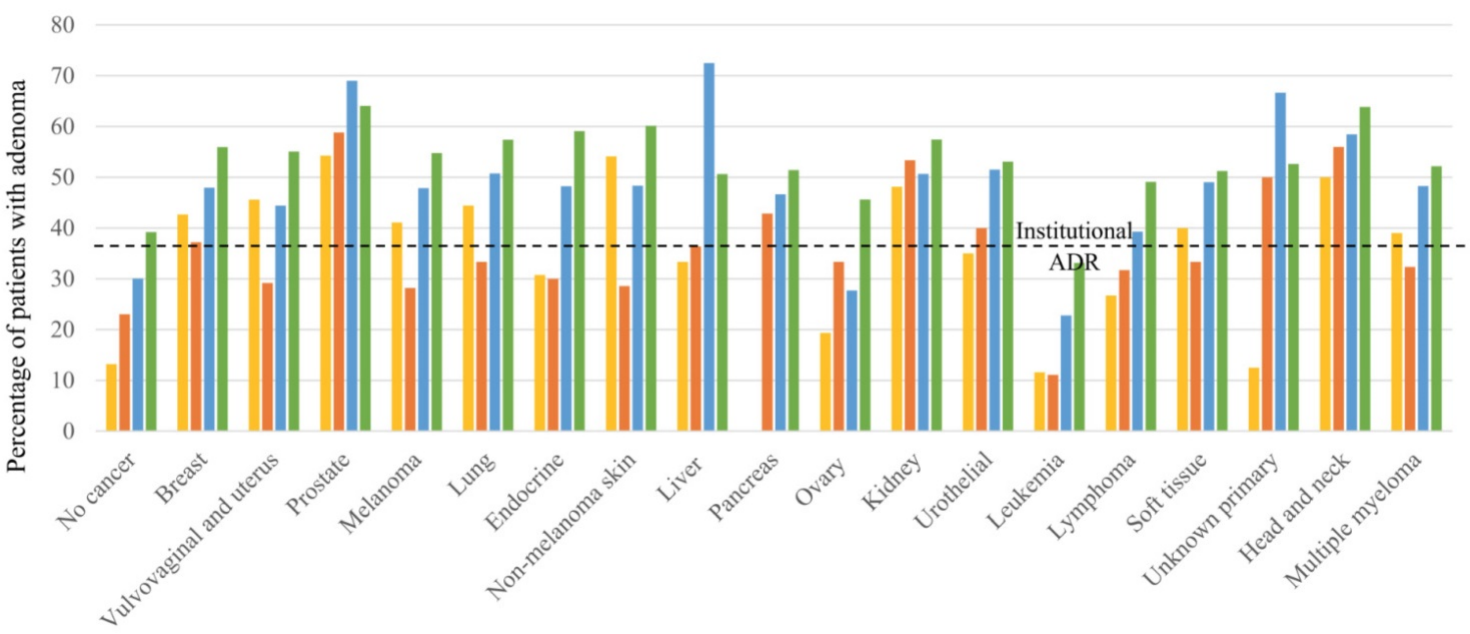
Adenoma detection rates in patients grouped by age and cancer type.

The numbers of patients and the ADR in different age groups stratified by cancer type are shown in **Supp. Table 1** and **Figure 1**. In patients younger than 40 years, the ADR was the highest in patients with prostate cancer (54.3%) and non-melanoma skin cancer (54.1%), followed by head and neck cancer (50.0%). In patients between ages 41 and 50 years, those with prostate cancer had the highest ADR (58.8%), followed by those with head and neck cancer (56.0%) and kidney cancer (53.3%). In patients between ages of 51 and 60 years, those with liver cancer had the highest ADR (72.5%), followed by patients with prostate cancer (69.0%) and those with cancer of an unknown primary source (66.7%). In patients older than 60 years, those with prostate cancer (64.1%) and head and neck cancer (63.9%) had the highest ADRs.

### Histologic features of adenoma

**Figure 2:**
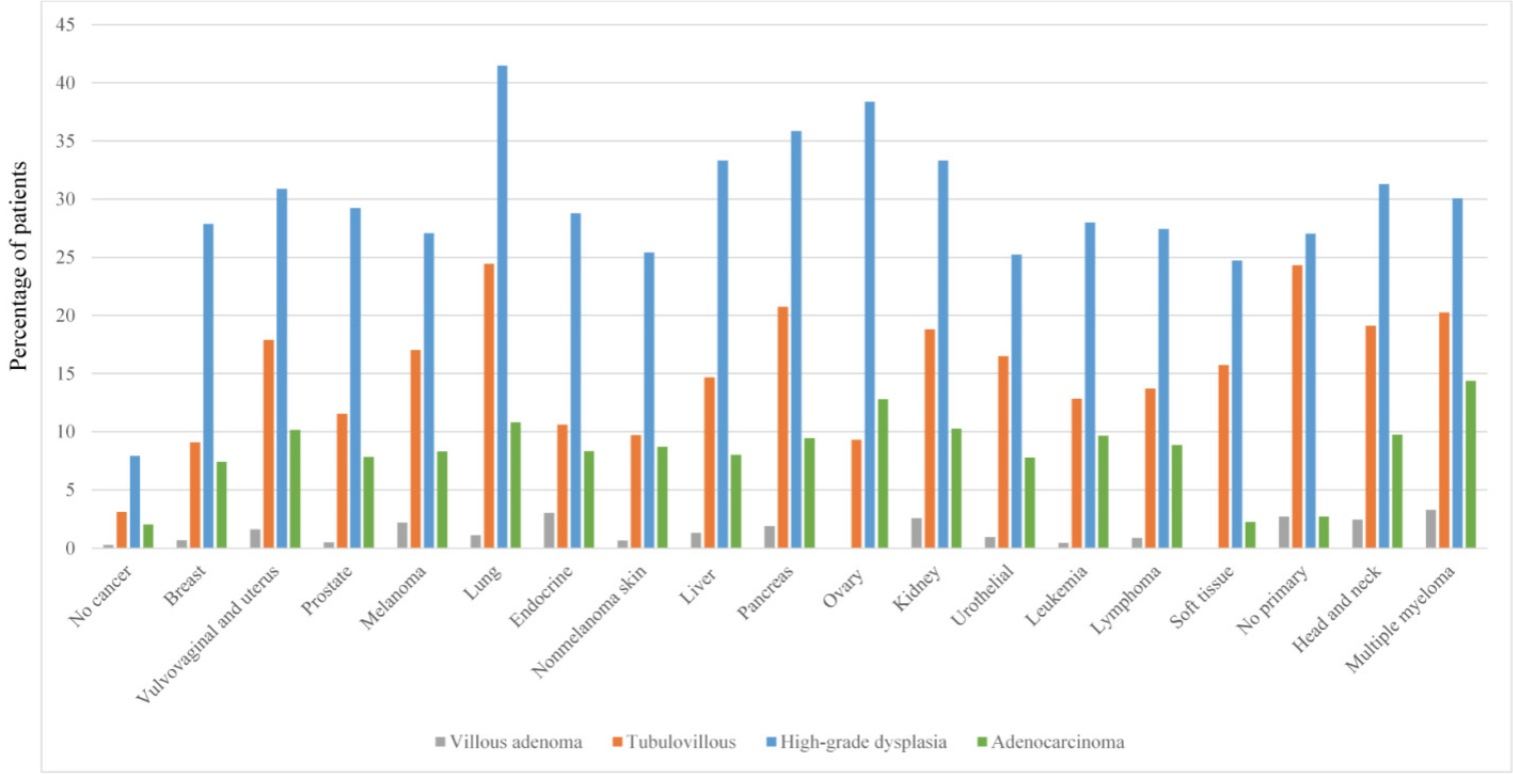
Advanced adenoma features grouped by cancer type.

Overall, high-grade dysplasia was reported in 1,611 patients (12.7%) whereas 620 (4.9%) patients had tubulovillous adenomas and 153 (1.2%) had villous adenomas. High-grade dysplasia was most commonly detected in patients with lung cancer (41.5%; **Figure 2**), followed by ovarian cancer (38.4%). Adenocarcinoma was diagnosed in 455 patients (3.6%). Of note, the highest proportion of adenocarcinoma detection was in patients with multiple myeloma (14.4%), followed by ovarian cancer (12.8%) and lung cancer (10.8%).

## Discussion

This study characterizes the incidence, histologic features, and risk of CAP in patients with a personal history of NCRC. Among patients with a history of NCRC, we found an ADR of 46%, with the highest rates observed among patients with a history of prostate cancer, non-melanoma skin cancer, head and neck cancer, and kidney cancer. The 46% ADR was greater than that of the control group or the general population at our institution. A low gastroenterologist turnover rate ensures consistency in our institutional ADR, and this consistency strengthens the implications of our findings. We found a personal history of NCRC to be an independent risk factor for CAP. With a 12.7% incidence of high-grade dysplasia and a 3.6% incidence of adenocarcinoma, our findings weigh in favor of lowering the age for initial CRC screening for NCRC patients.

Upon stratifying CAP incidence by age, we found that ADR tended to increase with age for most cancers. For individuals between 40 to 50 years with prostate cancer, the ADR was higher than it was for prostate cancer patients younger than 40 years. A similar trend of increased ADR with increasing age was observed among patients with pancreatic cancer, kidney cancer, urothelial cancer, head and neck cancer, and lymphoma. This trend highlights the impact of aging on the incidence of CAP and is in concordance with the significance of old age as an independent risk factor for the development of CAP. The high ADRs found among individuals younger than 50 years with certain cancers, such as prostate cancer and non-melanoma skin cancer, are concerning and warrant further investigation into the mechanisms behind the association between NCRC and CAP, which currently is a poorly studied issue. Additionally, the high ADRs among patients younger than 50 years highlight the importance of early screening colonoscopy among patients with a history of NCRC.

Previously reported studies regarding the risk of CRC are limited to certain types of NCRC and show inconsistent results 12,13. Little is known about the association between NCRC and CRC in the United States, and our study characterizes the incidence of CRC among a sample of American patients with a history of NCRC. We found advanced adenocarcinoma in 3.6% of the patients, with the highest incidence in patients with multiple myeloma, followed by ovarian cancer and lung cancer. CRC and NCRC share a considerable number of risk factors to which their association can be attributed.

One important component implicated in the cascade of events that lead to oncogenesis in CRC is the plethora of genetic mutations, some of which can also be present in patients with NCRC. The mutation in *APC*, a tumor suppressor gene that is a hallmark of some CRCs, was reported by Liberman and colleagues to be detected in NCRC as well 22. Likewise, a mutation in the *KRAS* proto-oncogene facilitates the second step in the carcinogenesis of 1 subtype of CRC.^23^ This alteration plays a pivotal role in some NCRCs' pathogenesis as well 24-26. Mutations in the *TP53* and *BRAF* genes have been implicated in the development of CRC as well as NCRC 27-30.

Associations between CRC and breast, ovarian, and uterine cancers have been reported in previous studies 31-35. Hormonal influences have been suggested to explain these observed associations, though it is not evident whether these hormonal influences act similarly in the context of CRC as they do in the development of breast, ovarian, and uterine cancers 36-38. The potential role of hormonal influences is further strengthened by the reported mitigation of CRC risk among patients receiving hormone replacement therapy, especially after menopause, and patients receiving estrogen receptor modulators 39-42.

Among the more established risk factors for CRC are obesity, old age, physical inactivity, dietary habits, alcohol use, and smoking 10,43. Cancer treatments such as chemotherapy and radiotherapy have also been implicated in the development of CRC 44-49. These risk factors can also predispose patients to a variety of NCRCs and may explain the commonality among these cancers. Intuitively, the association between NCRC and CAP may be more obvious in patients with a higher risk factor burden. Our analysis found a higher BMI to be associated with CAP detection. However, smoking was not found to have an independent association with CAP detection. The retrospective nature of our study limited our ability to analyze and account for additional known risk factors for CRC and NCRC. This highlights the need for efforts to analyze the factors potentiating the risk of CRC in patients with NCRC.

Our study has noteworthy limitations. First, the retrospective nature of our analysis limits the accuracy of our data. Second, our results might not be generalizable as they were based on data from a single center. Third, some of the colonoscopies in our study had been done for indications other than screening; this could affect the ADR. Fourth, we grouped all cancer types together in the multivariate analyses, and a separate analysis for each individual cancer type was not performed. This grouped analysis allowed us to characterize the CAP incidence rate but limited our ability to make recommendations regarding the appropriate timing for screening colonoscopy for specific cancer types. Since it is evident that the ADR is higher among certain cancers, the appropriate timeframe for screening colonoscopy after the diagnosis of each NCRC needs to be outlined in future studies. Last, although our study had a large sample size, in certain subgroup analyses our samples were underpowered.

## Conclusions

Our analysis revealed that patients with a personal history of NCRC had a higher ADR than did patients without a history of NCRC. The ADR is higher in certain cancers, including prostate cancer, head and neck cancer, kidney cancer, and non-melanoma skin cancer. The higher risk in these NCRCs warrants further studies of these individual cancers separately. Patients with a history of NCRC may benefit from screening for CRC earlier than 50 years of age. Future large-scale studies stemming from national or multicenter databases are warranted to further validate our findings and to investigate the appropriate timing for CRC screening as well as the appropriate interval for follow-up colonoscopy.

## Supplementary Material

Supplementary table.

## Figures and Tables

**Table 1:** Characteristics of patients with and without a history of noncolorectal cancer at the time of first colonoscopy

Characteristic	Cancer, No. (%) (n = 9408)	No Cancer, No. (%) (n = 3295)	*P*
Age, y, mean (SD)	58 (13)	54 (11)	< 0.001
Male sex	4401 (46.8)	1307 (39.7)	< 0.001
Race^a^			< 0.001
White	6462 (68.7)	2201 (67.0)	
Black	966 (10.3)	399 (12.1)	
Hispanic	949 (10.1)	189 (5.8)	
Asian	401 (4.3)	364 (11.1)	
Other	630 (6.7)	133 (4.0)	
Family history of colorectal cancer	1518 (16.1)	989 (30.0)	< 0.001
Smoking history	3953 (42.0)	800 (24.3)	< 0.001
BMI at time of colonoscopy, kg/m^2^, mean (SD)^b^	29.5 (6.6)	29.2 (6.4)	0.120

^a^Available for only 3286 patients in No cancer group; ^b^Available for 9,752 patients; Abbreviations: SD, standard deviation; BMI, body mass index.

**Table 2:** Characteristics of patients with and without adenomatous polyps detected on colonoscopy

Characteristic	Adenoma, No. (%) (n = 5453)	No Adenoma, No. (%) (n = 7250)	*P*
Age, y, mean (SD)	60 (12)	55 (13)	< 0.001
Male sex	2785 (51.1)	2923 (40.3)	< 0.001
Race^a^			0.025
White	3756 (68.9)	4907 (67.7)	
Black	610 (11.2)	755 (10.4)	
Hispanic	470 (8.6)	668 (9.2)	
Asian	291 (5.3)	474 (6.5)	
Other	324 (5.9)	439 (6.1)	
Family history of colorectal cancer	1084 (19.9)	1423 (19.6)	0.736
Smoking history	2183 (40.0)	2570 (35.4)	< 0.001
BMI at time of colonoscopy, kg/m^2^, mean (SD)^b^	30.0 (6.6)	28.9 (6.6)	< 0.001
Cancer			< 0.001
Yes	4503 (82.6)	4905 (67.7)	
No	950 (17.4)	2345 (32.3)	
Cancer type			< 0.001
Breast	1012 (22.5)	1028 (21.0)	
Prostate	780 (17.3)	436 (8.9)	
Lymphoma	452 (10.0)	650 (13.3)	
Leukemia	218 (4.8)	748 (15.2)	
Non-melanoma skin	299 (6.6)	258 (5.3)	
Vulvovaginal and uterus	246 (5.5)	281 (5.7)	
Melanoma	229 (5.1)	244 (5.0)	
Head and neck	246 (5.5)	165 (3.4)	
Lung	176 (3.9)	147 (3.0)	
Endocrine	132 (2.9)	152 (3.1)	
Multiple myeloma	153 (3.4)	170 (3.5)	
Kidney	117 (2.6)	101 (2.1)	
Urothelial	103 (2.3)	100 (2.0)	
Soft tissue	89 (2.0)	104 (2.1)	
Ovary	86 (1.9)	161 (3.3)	
Liver	75 (1.7)	62 (1.3)	
Pancreas	53 (1.2)	63 (1.3)	
Unknown primary	37 (0.8)	35 (0.7)	

^a^Information for race was not available in 2 patients in Adenoma group, and 7 patients in No adenoma group; ^b^Available for 9,752 patients; Abbreviations: SD, standard deviation; BMI, body mass index.

**Table 3:** Multivariate logistic regression for adenoma detection.

	Odds Ratio	95% Confidence Interval	*P*
Age	1.03	1.03 - 1.04	< 0.001
Male sex	1.49	1.37 - 1.62	< 0.001
Family history of colorectal cancer	1.38	1.25 - 1.54	< 0.001
Smoking history	0.97	0.89 - 1.05	0.426
BMI	1.03	1.02 - 1.03	< 0.001
Cancer	2.04	1.84 - 2.26	< 0.001

Abbreviation: BMI, body mass index.
