# Supplementary material for: Patients with non-colorectal cancers may be at elevated risk of colorectal neoplasia
Source: J Cancer. 2020 Mar 4;11(11):3192–8. doi: 10.7150/jca.40724 (PMC7097953; doi:10.7150/jca.40724)
Supplement: Supplementary file 1 — Supplementary table. [file jcav11p3192s1.pdf]

Supplemental Table 1. Number of patients in each age group by cancer status and type.

Cancer Type	Age				
	**≤ 40 y**	**41-50**	**51-60y**	**> 60 y**	**Total**
Breast	232	172	832	804	**2040**
Prostate	94	34	242	846	**1216**
Lymphoma	146	145	318	493	**1102**
Leukemia	224	117	272	353	**966**
Non-melanoma skin	61	35	180	281	**557**
Vulvovaginal and uterus	68	72	180	207	**527**
Melanoma	73	39	140	221	**473**
Head and neck	54	25	130	202	**411**
Multiple myeloma	41	34	87	161	**323**
Lung	27	6	67	223	**323**
Endocrine	52	30	114	88	**284**
Ovary	31	30	83	103	**247**
Kidney	27	15	75	101	**218**
Urothelial	20	5	33	145	**203**
Soft tissue	35	27	51	80	**193**
Liver	9	11	40	77	**137**
Pancreas	9	7	30	70	**116**
Unknown primary	8	8	18	38	**72**
Total (cancer)	**1211**	**812**	**2892**	**4493**	**9408**
No cancer	410	395	1614	876	**3295**
**Total**	**1621**	**1207**	**4506**	**5369**	**12703**
